# Affective Response to a Loved One's Pain: Insula Activity as a Function of Individual Differences

**DOI:** 10.1371/journal.pone.0015268

**Published:** 2010-12-16

**Authors:** Viridiana Mazzola, Valeria Latorre, Annamaria Petito, Nicoletta Gentili, Leonardo Fazio, Teresa Popolizio, Giuseppe Blasi, Giampiero Arciero, Guido Bondolfi

**Affiliations:** 1 Istituto di Psicologia Post-Razionalista IPRA Rome, Rome, Italy; 2 Department of Neurological and Psychiatric Sciences, University of Bari, Bari, Italy; 3 Institute of Psychiatry and Clinical Psychology, Department of Medical Sciences, University of Foggia, Foggia, Italy; 4 National Child and Deaf Family Service, South West London and St George's NHS Trust, London, United Kingdom; 5 Psychiatric Neuroscience Group, Section on Mental Disorders, Department of Neurological and Psychiatric Sciences, University of Bari, Bari, Italy; 6 Department of Neuroradiology, IRCCSS “Casa Sollievo della Sofferenza”, San Giovanni Rotondo, Italy; 7 Departement de Psychiatrie, Hopitaux Universitaires de Geneve, Geneve, Switzerland; The University of Melbourne, Australia

## Abstract

Individual variability in emotion processing may be associated with genetic variation as well as with psychological predispositions such as dispositional affect styles. Our previous fMRI study demonstrated that amygdala reactivity was independently predicted by affective-cognitive styles (phobic prone or eating disorders prone) and genotype of the serotonin transporter in a discrimination task of fearful facial expressions. Since the insula is associated with the subjective evaluation of bodily states and is involved in human feelings, we explored whether its activity could also vary in function of individual differences. In the present fMRI study, the association between dispositional affects and insula reactivity has been examined in two groups of healthy participants categorized according to affective-cognitive styles (phobic prone or eating disorders prone). Images of the faces of partners and strangers, in both painful and neutral situations, were used as visual stimuli. Interaction analyses indicate significantly different activations in the two groups in reaction to a loved one's pain: the phobic prone group exhibited greater activation in the left posterior insula. These results demonstrate that affective-cognitive style is associated with insula activity in pain empathy processing, suggesting a greater involvement of the insula in feelings for a certain cohort of people. In the mapping of individual differences, these results shed new light on variability in neural networks of emotion.

## Introduction

An important goal of integrating psychology and neuroimaging is to understand the detailed mechanisms mediating inter-individual differences in human behavior. Individual variability in emotion processing may be associated with genetic variations as well as with psychological predispositions [1]–[3]. In order to refine the integration between genetics and psychology, more psychological tools would be useful in the attempt to grasp the complexity of human variability. Several authors consider the dispositional affect to be the predominant modality of emotional engagement with the self and with the environment [4]–[10]. Our concept of dispositional affect developed based on previous works on the relationship between cognitive styles and attachment patterns [11]–[15] in some psychopathological conditions [16]–[17]. It emphasizes the need to account for the way in which each person, in dealing with others and the different circumstances of everyday life, feels situated in the environment [18]–[20]. Within this new perspective, two general dispositional affects can be defined: primarily based on basic emotions or primarily based on emotions which are co-perceived through others (non-basic emotions) [18]–[20]. Subjects with a better knowledge of basic emotions are said to have an inward disposition (not to be confounded with introversion-see below) [2]–[3], [21]–[22]. Inward subjects tend to be more viscerally aware, more sensitive in the detection of changes in bodily states occurring during emotions and feelings. In brief, their focus is primarily directed towards a frame of reference that predominantly uses a body-centered coordinate system [23]–[25]. Subjects with a better knowledge of non-basic emotions, i. e. emotions that require some kind of relationship between the self and external points of reference, are said to have an outward disposition (not to be confounded with extraversion -see below) [2]–[3], [26]. Outward subjects tend to be more externally aware, and in this sense, their focus is primarily directed towards a frame of reference that predominantly uses an externally-anchored coordinate system, i. e. contexts, people or rules and norms [27]–[31].

Different dispositional affects could explain behavioral data on field-dependent or independent perceptual processing [32], on independent or interdependent self-construal [31], [33], as well as on variability of interoceptive sensitivity in emotional processing [34]–[35].

Within these two general dispositional affects, five categories are identified as affective-cognitive styles among which two are particularly orthogonal: 1) phobic prone individuals (inward), and 2) eating disorders prone individuals (outward). It is necessary to underline that the terms phobic prone and eating disorders prone do not necessarily imply that these subjects are at higher risk of pathological phobias or of eating disorders. Phobic prone individuals rely predominantly on basic emotions and are characterized by a sense of permanence of Self predominantly centered on the visceral reading of emotional states. The “hypercognition” [21] of basic emotions (especially fear) plays a central role in the development and regulation of a stable perception of Self. In fact, the recurrent activation of basic emotions is matched by a subjective experience of “gut feelings”. As a result these individuals tend to regulate their relationship both with others and in accordance with the ongoing situations through bringing internal states into focus, thereby allowing their personal stability to coincide with the stability of their own bodily conditions (body-bounded sense of self). Therefore the bodily-emotional condition and its control (through various strategies) are centrally salient to these individuals in regulating their emotional life. On the other hand, eating disorders prone individuals are characterized by a sense of permanence of Self that emerges simultaneously and in tune with the perception of a source of meaning [28], [36]–[37]. While this produces a recognition of one's own internal states stemming from a focus on the real or imagined other in ongoing situations [29], [38]–[39], at the same time the “outward” referent becomes the source of information (perceived as source of expectations, of judgment, of emulation or as a pole of opposition, of challenge, etc.) to recognize one's own emotional experience. In this regard, eating disorders prone individuals tend to be more socially aware and to regulate their personal stability on a coordinate system which is outwardly anchored to a real or imagined other. One effect of this situational and social interest is that those emotions which emerge through mediated affective engagement (non basic emotions) can change more easily -since they tax the system's visceral resources less– and with greater flexibility with regard to the flow of ongoing events.

The insula plays a key role in homeostatic afferent activity that engenders distinct subjective bodily feelings [40], and it is involved in human feeling processing [41]–[44]. Therefore, its reactivity could be associated with individual differences. Neuroimaging studies have provided evidence for the direct involvement of the insular cortex in the so-called “pain matrix” during empathy for pain [45]–[53]. However, current debates on empathy have raised unanswered questions about individual differences [54]–[55]. To our knowledge, no fMRI studies have been conducted previously with this objective. Therefore, the present study was carried out to determine whether and how individual differences in affective-cognitive styles are associated with insula reactivity during affective empathic responses to directly perceived feelings of others.

With this objective, two groups of healthy subjects were categorized according to their affective-cognitive style, phobic prone or eating disorders prone. In order to study the role of the affective-cognitive styles, salient visual stimuli depict a loved one, in both painful and neutral expressions. Unfamiliar faces were used as controls. We predicted greater insular cortex activity associated with the subjective evaluation of their condition [40] in the phobic prone subjects, because of their relatively greater focus on a body-centered coordinate system as a frame of reference. More precisely, we hypothesized that the activation of the insular cortices during a visual experience of a loved one's pain would differ according to group.

## Materials and Methods

### Participants

Fifteen participants were phobic prone (PP) (6 females; mean age 39.2; standard deviation [SD] 7.4) and 15 were eating disorders prone (EDP) (5 females; mean age 34.4; standard deviation [SD] 8.65). The couples enrolled had been together in a committed relationship for the last three years and had been living together for at least one year. To assign the participants to a group, they were assessed with a semi-structured interview [2]–[3] and the Personality Meaning Questionnaire (PMQ) [56] one month before the scanning session. Concordance between the two investigators was 100%. As in our previous study [2]–[3], the semi-structured interview was administered independently by two trained investigators who were blind to each other's results. The aim of the semi-structured interview was to assess the key themes characterizing different affective-cognitive styles in the matter of emotional activation, duration and regulation. The semi-structured interview was divided into three consecutive steps. The subject was asked to give a detailed account of two meaningful emotional experiences involving anger and fear. After the account, the interviewer marked the characteristics of the appraisal, regulation and duration of the emotional experiences. If necessary, the interviewer asked for more details and then, the interviewer assessed the underlying predominant affective-cognitive style. The PP key themes detected were the tendency a) to focus on the visceral bodily states once the basic emotions have been triggered (automatic appraisal), b) to have the subjective perception of inability to modify these emotions after they have been triggered (duration), c) to have control over bodily-emotional condition aimed at limiting the emotional intensity; fear in these subjects lasts just as long as the perception of not being in control (regulation). Instead, EDP key themes detected were the tendency a) to focus on the outward referent recognized as the source of one's own emotional states (reflective appraisal), b) to have the subjective perception of capability to rapidly change these emotions by modifying the simultaneous focus on a different point of reference (duration), c) to adjust their personal stability to the perceived source of reference; being without a point of reference is perceived by these subjects as a feeling of emptiness (regulation). The PMQ questions on which PP subjects tend to score higher identify a score of need for emotional over-control in situations that may be felt as potentially dangerous (PP score) [56]. The questions on which EDP subjects score higher identify a score for need for consent and approval, sensitivity to judgment, and vulnerability to criticism (EDP score) [56].

A behavioral evaluation of how subjects in each group processed empathy for pain was obtained through the Interpersonal Reactivity Index (IRI) [57] comprised of four subtests which measure dispositional empathy based on the notion that empathy consists of a set of separate but related constructs. In order to support the behavioral characterization of each dispositional affect style in terms of body perception, we also employed two subtests of the Body Perception Questionnaire (BPQ) [58]: the “Awareness of Bodily Processes” (ABP) and the “Autonomic Nervous System Reactivity” (ANSR). The study was approved by the local IRB. Subjects also completed a series of questionnaires identifying different personality characteristics, such as the NEO Five Factors Inventory [59], the Temperament and Character Inventory (TCI) [60], the Positive and Negative Attitude Scale (PANAS) [61], the Eysenck Personality Inventory (EPI) [62], and the Big Five Questionnaire (BFQ) [63]. Other demographic variables included years of education, parental socioeconomic status [64], total IQ (assessed with the Wechsler Adult Intelligence Scale-Revised [WAIS-R]), and handedness [65] (Table 1). Exclusion criteria included a history of drug or alcohol abuse, previous head trauma with loss of consciousness, pregnancy, and any significant medical or psychiatric conditions as evaluated with the SCID interview.

**Table 1:** Questionnaire Scores for Phobic prone and Eating disorders prone Groups.

		PHOBIC PRONE(PP) n = 15		EATING DISORDERS PRONE (EDP) n = 15	
Questionnaires	t value	Mean	SD	Mean	SD
**IRI**					
Perspective Taking	t = −3.65 p<0.001	21	4.63	26	3.13
Fantasy	t = −1.50 p>0.14	21	4.34	24	4.17
Empathic Concern	t = −1.01 p>0.3	26	2.55	27	3.81
Personal Distress	t = 0.80 p>0.43	17	6.20	16	2.69
**Body Perception Questionnaire**					
Awareness of Bodily Processes	t = 2.6 p<0.03	2.41	1.06	2.25	0.7
Autonomic Nervous System Reactivity	t = 1.39 p>0.10	1.68	0.44	1.39	0.48
**Positive and Negative Attitude Scale**	t = 1.4 p>0.17				
Positive		33.1	3.4	32.0	8.7
Negative		19.1	9.0	20.0	7.2
**Eysenck Personality Inventory**	t = 0.8 p>0.4				
Psychoticism		3.2	2.2	5.0	3.2
Extraversion		14.4	4.2	13.9	3.2
Neuroticism		8.7	4.9	9.8	5.8
**NEO Five Factors Inventory**	t = 0.5 p>0.62				
Neuroticism		19.9	6.6	21.2	5.4
Extraversion		30.8	6.3	28.0	4.7
Openness		29.6	4.4	31.6	4.1
Agreeableness		29.0	4.7	31.1	6.4
Conscientiousness		31.3	6.3	29.6	5.7
**Temperament and Character Inventory**	t = 1.67 p>0.11				
Harm avoidance		9.01	3.5	9.6	4.1
Novelty seeking		9.5	3.8	10.2	3.9
Reward dependence		10.2	6.5	9.3	3.2
Persistence		2.4	1.7	1.8	1.4

Underlined rows report significant differences between the PP and EDP groups. SD = standard deviation.

### Ethics statement

The present study was approved by the Comitato Etico Indipendente Locale of the Azienda Ospedaliera “Ospedale Policlinico Consorziale” of Bari. Informed written consent was obtained from all participants before participation.

### Functional MRI data

fMRI data were acquired on a 3T GE (General Electric, Milwaukee, WI) MRI scanner with a gradient-echo echo planar imaging (EPI) sequence and covered 26 axial slices (5 mm thick, 1 mm gap), encompassing the entire cerebrum and most of the cerebellum (TR 2; field of view, 24 cm; matrix, 64×64, a voxel size of 3.75×3.75×5 mm). For each scan, a total of 330 EPI volume images were acquired.

### Visual Stimuli

**Figure 1:**
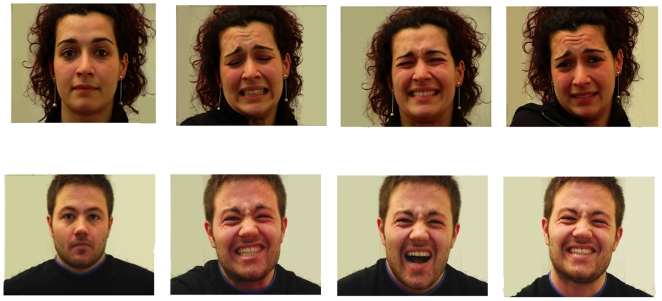
Sample of visual stimuli: actors' neutral and painful facial expression.

Visual stimuli consisted of 160 pictures (720×576 pixels), 40 for each condition, depicting faces of a loved one and of actors, in both painful and neutral situations. Two professional actors, a female and a male, were enrolled as models for the pictures of unfamiliar faces (Figure 1). Facial expressions of actors and partners were filmed in a session previous to scanning. Painful facial expressions were elicited by mechanical stimuli during a pain threshold test. Two investigators reviewed the videotaped recordings and selected by consensus the picture frames conveying evidence of the intensity of the experience of pain, based on Ekman and Friesen's Facial Action Coding System (FACS) [66].

### General fMRI Procedures

Functional MRI scanning consisted of one run in an event-related design. To optimize the stimulus sequence, we used a genetic algorithm [67]. The exact timing of the occurrence of each event was generated with the genetic algorithm, using an average inter-stimulus interval (ISI) of 1300 ms, equal numbers of on and off events, and optimization for hemodynamic response detection. Visual stimuli were presented for 1400 ms in a random order. During the interstimulus interval (ISI), a crosshair was presented. Total run time was about 11.2 minutes. Visual stimuli were presented using Presentation 10.5 (www.neuro-bs.com). During the scanning session participants were required to perform a discrimination task between known and unknown faces, in both painful and neutral situations. Responses were given via a button box which recorded accuracy (i. e. percent correct responses) and reaction time (measured in milliseconds). Before the scanning session, each participant completed the STAI questionnaire [68] to evaluate their state of anxiety. After scanning, participants were asked to rate the intensity of others' pain and of their own feelings of unpleasantness on the basis of the same visual stimuli by using a computerized visual analogue scale (VAS) with target words ranging from “no pain” to “extreme pain” and from “no effect” to “extreme unpleasantness”. Participants were not informed of their partners' role in the study before the scanning session.

### Image analysis

Images were preprocessed and analyzed using SPM5 (Wellcome Department of Cognitive Neurology, London, UK), implemented in MatLab 7.2 (MathWorks™). For each subject, functional images were first slice-timing corrected, using the middle slice acquired in time as a reference, and then spatially corrected for head movement, using a least-squares approach and six-parameter rigid body spatial transformations. They were then normalized into a standard stereotactic space (Montreal Neurological Institute MNI template) by using a 12-parameter affine model and spatially smoothed with a three-dimensional Gaussian filter (10 mm full-width at half-maximum).

Images were analyzed using a standard random-effect procedure. The time series of functional MR images obtained from each participant were analyzed separately. The effect of the experimental paradigm was estimated on a voxel-by-voxel basis, according to the general linear model extended to allow the analysis of fMRI data as time series. Low-frequency noise was removed with a high-pass filter (time constant 128 s). The onset of each trial constituted a neural event that was modeled through a canonical hemodynamic response function, chosen to represent the relationship between neural activation and hemodynamic changes. Serial correlation in the fMRI time series was estimated with a restricted maximum likelihood (ReML) algorithm using an autoregressive AR(1) model during parameter estimation, assuming the same correlation structure for each voxel. The ReML estimates were then used to whiten the data. These subject-specific models were used to compute four contrast images per subject (partner's neutral face, partner's painful face, unknown neutral face, unknown painful face), each representing the estimated amplitude of the hemodynamic response in one experimental condition. Contrast images from all subjects of the two groups (inward and outward) were entered at the second level into a random-effects model repeated-measures 2×2×2 ANOVA with non-sphericity correction (as implemented in SPM5). For interaction analyses and direct comparisons of the two groups a 2×2×2 factorial design was used: a group factor (inward-outward), a painful facial expressions factor (painful-neutral faces) and a “familiar” facial expressions (partner's-unfamiliar faces). Across all analyses, the statistical threshold was set at p<0.001 uncorrected with an extent threshold of 8 contiguous voxels. Fisher's LSD test was used for post-hoc comparisons. All MNI coordinate spaces were converted to the Talairach coordinate system by icbm2tal (http://brainmap.org/icbm2tal/).Anatomic and Brodmann's areas labeling of the activity of clusters was performed with the Talairach Daemon database (http://www.talairach.org/).

In order to investigate signal intensity of BOLD responses, regions-of-interests (ROIs) were defined as spheres with 6 mm diameter centered at the peak voxel in the activated clusters identified in the 3-way interaction analysis. The parameter estimates of signal intensity in ROIs were computed from the first-level analysis in each participant and successively compared with a repeated measures ANOVA, with four facial expressions as within-effect factors and with dispositional affects as between-subjects factors.

In order to evaluate any differences between groups for VAS ratings intensity of the others' pain and of their own feelings of unpleasantness, a 2×2×2 factorial design was used with the group factor (PP-EDP), pain factor (painful-neutral faces) and familiarity factor (partner's-unknown faces). T tests were used to verify any difference s between groups due to the familiarity factor in VAS ratings of the intensity of others' pain and of their own feelings of unpleasantness. T tests were employed to evaluate any differences between groups in questionnaires. Repeated measures ANOVAs with dispositional affects as the between-subjects factor were carried out to analyze any differences in reaction time and performance accuracy.

## Results

### Demographics and questionnaires

T tests and χ^2^ indicated that the two groups of subjects were well matched for age, gender, parental education and years of education (all p>0.2). T tests of the IRI scores only revealed a significant difference between groups for one subtest, “Perspective Taking” (PT), which measures the reported tendency to spontaneously adopt the psychological point of view of others in everyday life (t-value  = −3.65 df = 28 p<0,001): the EDP group had higher PT scores than the PP group (Table 1). Interestingly, subjects in the PP group had higher scores than outward subjects for the “Awareness of bodily processes” (ABP) subtest (t-value  = 2.6 df = 28 p<0.03) (Table 1). These results provide evidence that the two groups have different questionnaire response rates: the PP group was more likely to be aware of bodily processes and a less prone to adopt another's point of view, whereas the opposite tendency was seen in the EDP group, i.e. more likely to adopt another's point of view and less likely to be aware of bodily processes. T tests of the other questionnaires did not indicate any significant difference between groups (df = 28; NEO: t-value = 0.5 p>0.62; TCI: t-value = 1.67 p>0.11; PANAS: t-value = 1.4 p>0.17; EPI: t-value = 0.8 p>0.4; BFQ: t-value = 1.96 p>0.06), suggesting that the two groups of subjects did not significantly differ on other aspects of personality identified by these questionnaires (Table 1).

On the other hand, the VAS ratings ANOVA revealed that no significant interactions occurred between the group factor, pain factor and familiarity factor, in both the evaluation of pain intensity in others and in the personal experience of unpleasantness when observing others' pain. No significant differences due to the familiarity factor were found between groups in VAS ratings of the intensity of others' pain or in participants' own feelings of unpleasantness.

In addition, in a repeated measures ANOVA with the dispositional affects factor as the between-subjects factor showed no differences between the two groups in terms of reaction time and performance accuracy.

### Neuroimaging Results

**Figure 2:**
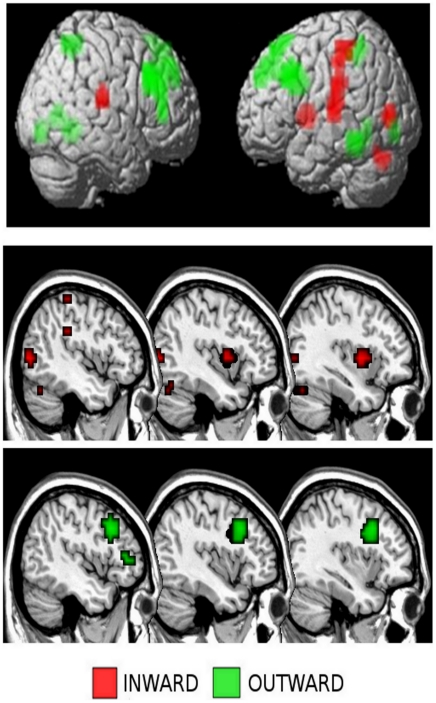
3D rendering (image threshold at p<0.05 FWE corrected) of the BOLD response of the main effects of group factor. Significant activation: INWARD: left posterior insula BA13, and the right parietal lobe SII BA40; OUTWARD: bilateral DLPFC BA9, bilateral precuneus BA7. 2-D overlay with multiple slices of insular activation in each group.

First of all, the main effects of pain, familiarity and affective-cognitive style factors were investigated. Observing pain in others (painful faces>neutral faces) caused activation in the right dorsolateral prefrontal gyrus (BA 46) (DLPFC), left cerebellum and right red nucleus (p<0.001 uncorrected) (Table 2). In contrast, the main effect of the familiarity factor [partner's faces>unknown faces] was associated with activation of the right inferior frontal gyrus (BA9), the right medial prefrontal cortex (BA10) and the left posterior cingulate cortex (BA31) (p<0.001 uncorrected) (Table 2). Previous studies have found these same areas to be involved in cognitive and emotional processing of pain empathy and familiarity. The main effect of the affective-cognitive style was interesting to observe, as the group factor produced a significant effect. Indeed, activity in the left posterior insula (BA13) and the right parietal lobe (BA40) (SI) (p<0.001 uncorrected) was greater in the PP group; whereas in the EDP group, the BOLD response was greater in the bilateral DLPFC (BA9), bilateral precuneus (BA7) and left posterior cingulate cortex (BA23) (PCC) (p<0.001 uncorrected) (Figure 2, Table 3). Interestingly, in the PP group, greater activation was seen in those areas usually involved in the bodily states, even though no real bodily experience was administered.

**Table 2:** Main effects of pain and familiarity factors p<0.001 uncorrected, k = 8.

		MNI coordinates				
Main effect	Region	x	y	z	k	Z Scores
Pain>Neutral	Right BA46 middle frontal gyrus	49	22	15	275	5.57
	Left BA9 middle frontal gyrus	−52	19	25	243	5.26
	Left anterior cerebellum	−45	−45	−30	46	4.66
	Right BA22 temporal gyrus	56	−45	−5	108	4.55
	Left BA38 superior temporal gyrus	−34	4	−30	46	4.52
	Right Amygdala°	26	−8	−25		3.9
	Right Midbrain red nucleus	8	−19	−15	41	4.32
Partner>Unfamiliar	Right BA9 inferior frontal gyrus	49	19	20	103	5.03
	Right BA10 medial frontal gyrus	8	71	5	249	4.44
	Left BA31 posterior cingulate cortex	−8	−52	30	188	4.42
	Left BA47 inferior frontal gyrus	−45	22	−20	74	4.07
	Left BA37 middle temporal gyrus	−45	−64	15	50	3.80
Unfamiliar> Partner	Left BA3 parietal gyrus	−38	−19	45	90	3.85

°same cluster.

**Table 3:** Inter-group comparisons p<0.001 uncorrected, k = 8.

		MNI coordinates				
Main effect of group	Region	x	y	z	k	Z Scores
Phobic prone >Eating disorders prone	Left BA13 insula	−38	0	5	105	6.53
	Left BA19 inferior occipital gyrus	−45	−79	5	29	6.53
	Left BA41 temporal transversus gyrus	−60	−22	10	276	6.22
	Right BA40 parietal lobe	68	−22	20	35	6.18
	Left Cerebellum posterior lobe	−30	−71	−25	80	5.59
Eating disorders prone >Phobic prone	Right BA9 middle frontal gyrus	49	22	35	554	6.47
	Left BA4 precentral gyrus	−26	−15	75	15	6.30
	Right cerebellum anterior lobe	15	−52	−5	506	6.27
	Left BA9 middle frontal gyrus	−45	19	35	182	6.19
	Left BA7 precuneus	−22	−45	50	56	6.15
	Left BA4 precentral gyrus	−64	−8	35	73	5.85
	Right BA18 lingual gyrus	4	−82	−5	153	5.85
	Left BA8 medial frontal gyrus	−8	49	35	24	5.68
	Left BA23 posterior cingulate cortex	0	−34	20	43	5.24
	Right BA7 precuneus	26	−49	55	21	5.14
3-way interaction	Left BA13 insula°	−41	−4	10	8	3.01
	Left BA31 precuneus	−26	−71	35	11	3.56
	Right BA10 medial frontal cortex	11	60	−5	8	3.11

°p<0.01 uncorrected, k = 8.

**Figure 3:**
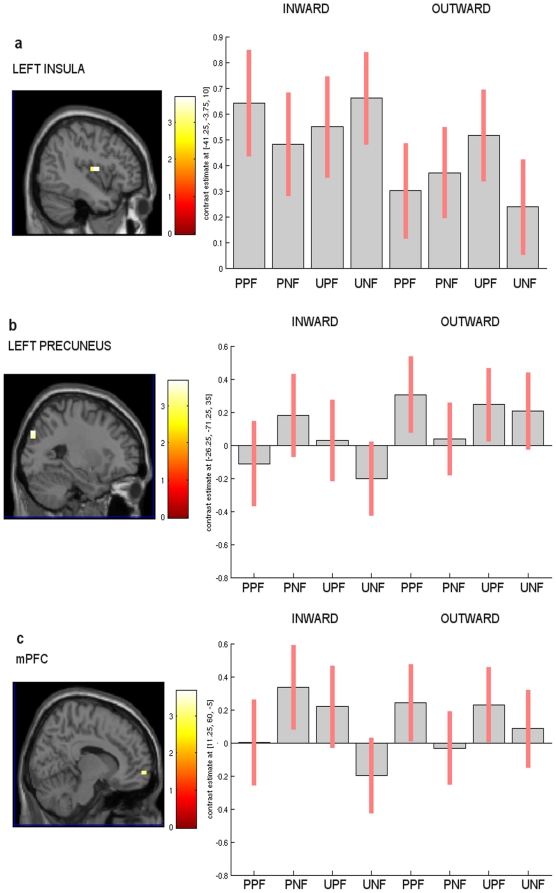
Left insula with significantly greater activation in the inward group, obtained with the the 3-way interaction analyses, identified at p<0.001 uncorrected, Ke = 8 voxels. **a**) the left posterior insula (peak coordinates x = −41, y = −4, z = 10; p<0.01 uncorrected, Ke = 8 voxels); **b**) the left precuneus (peak coordinates x = −26, y = −71, z = 35); **c**) the right medial PFC (peak coordinates x = 11, y = 60, z = −5). PPF (partner's painful faces), PNF (partner's neutral faces), UPF (unfamiliar painful faces), UNF (unfamiliar neutral faces). Bars depict variance loadings and 90% confidence intervals.

At this point, the three-way interaction between affective-cognitive style, the observed facial expression, and the familiarity of the face was explored. This interaction demonstrated differential activity in the left insula (BA13) (x = −41 y = −4 z = 10) at a more lenient threshold (p<0.01) (Figure 3a). Moreover, the interaction also indicated differential activity in left precuneus (BA31) (x = −26 y = −71 z = 35; p<0.001) (Figure 3b) and in the right mPFC (BA10) (x = 11 y = 60 z = −5; p<0.001) (Figure 3c, Table 3). ANOVA analyses of parameter estimates from these clusters indicated greater activity in the left insula for the PP group during processing of partners' painful expressions and of strangers' neutral expressions. On the other hand, in the EDP group, the left precuneus was more engaged and the right mPFC (BA10) was less deactivated during processing of partners' painful expressions and of strangers' neutral expressions (Figure 3a, 3b, 3c). This finding suggests that a significant correlation was present between the group factor and the differences observed among the participants.

## Discussion

The present study investigated the relationship between affective-cognitive styles and insula reactivity during affective empathic responses to the directly perceived feelings of others.

For this purpose, visual stimuli depicting partners' and unknown faces, in both painful and neutral situations, were presented to two groups of healthy participants, categorized according to their affective-cognitive style, inward (phobic prone) or outward (eating disorders prone).

The results suggest that affective-cognitive style is associated with differential insula reactivity to painful facial expressions. Imaging data revealed that the involvement of the insular region was quantitatively different in these two groups of healthy subjects categorized according to their affective-cognitive styles. Interaction analyses demonstrated that different brain regions were more involved in each group, particularly while processing partners' painful facial expressions. Indeed, in PP subjects, more activation was seen in the left posterior insula, whereas EDP subjects had greater engagement in the left precuneus and mPFC.

Up to the present, several studies have provided results that assign a key role to the insula during the direct experience of pain and during a vicarious experience of another person's pain [47]–[53], [69]–[74]. These studies significantly contributed to the development of the current topic concerning insular cortex engagement in emotions [47]–[50]. Concerning the individual differences issue in pain empathy [54]–[55], our results suggest that dispositional affects act upon the neural regions that subserve the ability to appreciate others' pain. At least two conclusions can be drawn from this fact. First, the role played by the insular cortex in the affective response to others' pain is central for a certain cohort of people. Secondly, the engagement of the insula in emotional experiences is modulated by dispositional affects.

A primary interoceptive representation of the physiological condition of the body has been shown to exist in the posterior insular cortex [40], [75]–[76]; thereby it is involved in human feelings [41]–[44]. As suggested by Craig's studies, a phylogenetically new homeostatic afferent pathway from lamina I, through the thalamus, to the posterior insular cortex provides a direct representation of homeostatic afferent activity that engenders distinct bodily feelings such as pain and visceral sensations [44], [76]. Thus, it seems that the regions with more activity in the PP group engage first-order mapping structures like the posterior insula and the somatosensory cortex/SI- recipients of signals from the internal milieu and the viscera [40], [44], [75]–[83]. These results are consistent with the theoretical construct of inwardness described by our model [18]–[20]. As they are more aware of the changes in bodily states occurring during emotions and feelings, these subjects had greater involvement of an area such as the posterior insula associated with regulating bodily states.

In contrast, the EDP group had greater activation in fronto-posterior parietal areas, such as the medial prefrontal cortex and the precuneus, while processing partners' painful facial expressions. The role of the precuneus has been demonstrated in processing self-relevant contextual information [84]–[88], in attention tracking [88]–[89], and in attentional non spatial shifts [90]. On the other hand, the mPFC/BA10 as a whole plays a role in self-referential processing [87], [91]–[94] and social cognition [39], [95]. It has also been suggested that the mPFC/BA10 influences the attentional balance between self-generated and perceptual information, rather than being exclusively involved in processing self-generated information [96]. Thus, the precuneus (and interconnected posterior cingulate) and medial prefrontal cortices are engaged in continuous information gathering and representation of the self and the external world (“co-perception”), as well as in the assessment of self-relevant sensations [87]–[88], [91], [97]–[98]. Such results seem to be consistent with our model's of outwardness, as these subjects preferentially use an externally-anchored coordinate system as a reference frame during emotions and feelings.

The greater response in the left insula in the PP group to unfamiliar neutral faces, and in the left precuneus and right mPFC in the EDP group, could be related to the processing of the emotional significance of ambiguous stimuli when making a judgment [99]. This speculation is supported by the observation that both groups had similar BOLD responses.

The activation of different regions for the two groups cannot be attributed to dispositional variables such as sensitivity to pain expression: the two groups did not differ in their ratings of the intensity of pain in others or in their personal feelings of unpleasantness when observing others' pain. Moreover, behavioral data support the inter-group differences: PP subjects scored higher on indexes of internal body perceptions on the on the “Awareness of Bodily Processes” questionnaire [58], while EDP subjects scored higher on indexes of “Perspective Taking” questionnaire [57].

It is interesting to note that our results are in line with those of Critchley's study [79] which used a synchronized or desynchronized heartbeat tone signal in contrast with a series of ten similarly timed tones that either did or did not include an oddball tone. In this study, the interaction between desynchronized timing and interoceptive attention highlighted several regions including the precuneus and posterior insula. These regions are directly involved in interoceptive attention and exteroceptive attention. Interestingly, the engagement of the precuneus was greater when one person was in a painful situation caused by another individual, that is to say, when attention was focused on the social context [100].

One limitation of this study was the insula sub-threshold activation reached in the interaction analysis. It should be viewed in the context of the significant main effect of the group factor.

In conclusion, all these findings indicate that affective-cognitive styles play a key role in explaining individual differences in insula reactivity when observing partners' painful facial expressions. As predicted, during cerebral processing of emotions, imaging in the PP group demonstrated a greater engagement of the posterior insula, which is involved in mapping of internal bodily and subjective feeling states. Evidence exists that the insula plays an important role in connecting emotional experience with interoceptive states [24]–[25], [82]–[83]. Beyond what was expected, imaging in the EDP group demonstrated activation in those regions that are engaged in continuously gathering and visualizing concurrent information on the self and the external world (co-perception).

Other than offering new insights into individual differences in the pain empathy issue, these new data shed new light on the variability in neural networks of emotion [44], and on the approach to the emotional embodiment issue [10], [101]–[104].
